# Dynamic comparison between Daan real-time PCR and Cobas TaqMan for quantification of HBV DNA levels in patients with CHB

**DOI:** 10.1186/1471-2334-14-85

**Published:** 2014-02-14

**Authors:** Shao-hang Cai, Fang-fang Lv, Yong-hong Zhang, Ye-gui Jiang, Jie Peng

**Affiliations:** 1 NanFang Hospital, Southern Medical University, Guangzhou, Guangdong Province, China; 2 Sir Run Run Shaw Hospital, Zhejiang University, Hangzhou, Zhejiang Province, China; 3 Second Xiangya Hospital, Central South University, Changsha, Hunan Province, China; 4 Southwest Hospital, the Third Military Medical University, Chongqing, China

**Keywords:** HBV DNA quantification, Daan Test, Cobas TaqMan, Hepatitis B virus, Viral load, Serum, HBeAg

## Abstract

**Background:**

Hepatitis B virus (HBV) DNA levels are crucial for managing chronic hepatitis B (CHB). It was unclear whether Daan real-time polymerase chain reaction test (Daan test) or COBAS TaqMan HBV DNA Test (Cobas TaqMan) was superior in measuring different HBV DNA levels in clinical specimens.

**Methods:**

We enrolled 67 treatment-naïve, HBV surface antigen-positive CHB patients (high baseline viral levels) who received either lamivudine/adefovir or entecavir. Serum samples were tested at baseline and treatment week 24 using the Daan test and Cobas TaqMan.

**Results:**

In the 67-baseline samples, the HBV DNA levels with the Cobas TaqMan (7.90 ± 0.73 log_10_ IU/mL) were significantly greater than those of the Daan test (7.11 ± 0.44 log_10_ IU/mL; *P* < 0.001). Of the 67 24-week samples (low viral levels), the Cobas TaqMan detected 59 (88.1%; 8 undetected); the Daan test detected 33 (49.3%; 34 undetected; *P* < 0.001). The Cobas TaqMan detected HBV DNA in 26 of 34 samples undetectable by the Daan test (range, 1.4–3.7 log_10_ IU/mL) or 38% of samples (26/67). The reductions in viral load after 24 weeks of oral antiviral treatment in the 33 samples that were positive for both the Daan test and the Cobas TaqMan test were significantly different (3.59 ± 1.11 log_10_ IU/mL versus 4.87 ± 1.58 log_10_ IU/mL, respectively; *P* = 0.001). Spearman correlation analysis showed positive correlation between results from two tests (r_p_ = 0.602,P<0.001). The HBV genotypes and the anti-viral treatment did not affect the measurements of the HBV DNA by the Daan assay and the Cobas Taqman assay.

**Conclusion:**

The Cobas Taqman was more sensitive at low viral loads than the Daan test and the change from complete to partial virological response could affect clinical decisions. The Cobas Taqman may be more appropriate for detection of HBV DNA levels.

## Background

Chronic hepatitis B (CHB) and its chronic sequelae (e.g., cirrhosis and hepatocellular carcinoma (HCC)) are major health problems, especially in Asia [1,2]. Detecting hepatitis B virus (HBV) DNA, a marker of viral replication, strongly indicates evidence of an HBV infection, as HBV surface antigen (HBsAg) is detected [3,4]. The detection of HBeAg is important, as its presence usually indicates active HBV replication and a higher risk for HCC [5]. Also, the presence of HBeAg may imply an earlier phase or an active infection with HBV [2].

A goal of CHB therapy is to persistently suppress HBV replication [6]. Efficacy of treatment for CHB ranges from 17% to 53% [7-9], and is hindered by the development of HBV resistance to ongoing therapy [10]. Measurement of HBV DNA levels is the most direct and effective way to assess HBV management and high HBV DNA levels are risk factors for development of fibrosis, cirrhosis, hepatocellular carcinoma, and drug resistance [11]. Since fluctuations in HBV DNA levels often trigger a modification in the patients’ treatment regimen, the accuracy and detection limits of the methods that quantitate HBV DNA levels can influence the clinical decision making process, including the dosage, the schedule, and subsequently monitoring of CHB patients [12-15]. Yuan and colleagues [13] found that only 38% of serum samples (161 of 425) of Chinese CHB patients had detectable HBV DNA levels by a liquid hybridization method (Digene Hybrid Capture II test (Digene test; Digene, Gathersburg, MD, USA); but HBV DNA levels were detected in 89% of these samples (383/425) by the real-time polymerase chain reaction (PCR) method, Cobas TaqMan assay (Cobas TaqMan, Roche Molecular Systems, Inc., Branchburg, NJ). Currently, real-time PCR quantification assays are used for measuring HBV DNA due to their sensitivity, specificity, and dynamic range [12,16,17]. Accuracy is enhanced when the HBV DNA is assayed within a linear range [3,13,16]. Comparison of the accuracy and detectable range between the different PCR tests suggest that the Cobas TaqMan assay (Cobas TaqMan) shows similar sensitivity and specificity rates to the RealArt assay (Abbott, North Chicago, IL USA) [3].

However, to our knowledge, comparisons between the Cobas TaqMan assay and the Daan test of HBV DNA levels have not been performed with clinical samples from patients with both high and low viral loads, particularly those from posttreatment patients who may have very low viral loads. In this multicenter, prospective, cohort study, we aimed to investigate any potential differences in detecting HBV DNA levels between the 2 methods in a clinical setting by testing samples with high or low viral loads taken from Chinese carriers at baseline and after 24 weeks of oral antiviral treatment.

## Methods

### Subjects

This study was a multicenter prospective cohort study. From July, 2011 to March, 2012, we enrolled a cohort of 67 treatment-naive patients (48 males, 19 females), who were diagnosed with hepatitis B virus e antigen (HBeAg)-positive CHB with high viral loads at baseline. Of the 67 patients, eight patients were from Southwest Hospital, six patients were from Sir Run Run Shaw Hospital, eighteen patients were from Second Xiangya Hospital, and the remaining thirty-five patients were from NanFang Hospital. The inclusion criteria were HBeAg-positive CHB with high viral loads at baseline (HBsAg positive >6 months; serum HBV DNA levels >2 × 10^6^ log_10_ IU/mL by the Daan test). HBeAg was positive by radioimmunoassay (Abbott Laboratories). We also included patients who had alanine aminotransferase (ALT) levels greater than the upper limit of normal (ULN), documented on 2 separate occasions 2 weeks apart, with the latest ALT ≥ 2 × ULN. Exclusion criteria were evidence of cirrhosis or coinfections with any other viruses, such as hepatitis C virus or human immunodeficiency virus (HIV), or presence of autoimmune hepatitis, alcohol liver disease, or hepatocellular carcinoma.

The Institutional Review Board of Nanfang Hospital, Southern Medical University of the participating sites had approved the study to compare the sensitivity and range of the two assays, Daan test and Cobas PCR test. Each enrolled patient provided informed consent. Patients were treated with entecavir (ETV) or the combination lamivudine (LAM) plus adefovir (ADV), according to the Chinese guidelines; tenofovir disopril fumarate was not available in China. Because patients may have used economic considerations to choose drug treatment, the drug treatment was not randomized. Forty-one (61.2%) of the sixty-seven patients received oral antiviral treatment with LAM plus ADV combination therapy, and the remaining twenty-six patients received entecavir (ETV) monotherapy. Blood samples were obtained at baseline and at week 24 and serum samples were collected. All samples were aliquoted and stored immediately at –80ºC.

### Quantification of serum HBV DNA and determination of HBV genotype

The Daan test was performed at the Nanfang Hospital per the manufacturer’s instructions (Daan Gene Co, Ltd of Sun Yat-sen University, Guangdong, China). Briefly, HBV DNA was extracted with control DNA from 100 μL of serum and detected by PCR fluorescence probing. The real-time PCR assay was performed in the Roche LightCycler 480 (Roche Diagnostics Ltd,Switzerland) according to the manufacturer’s instructions; the PCR amplification reaction used thermus aquaticus DNA polymerase, deoxyribonucleoside triphosphates (dNTPs), and a HBV DNA specific probe to detect and measure serum HBV DNA levels. The manufacturer reports a linear range of 1 × 10^2^ IU/mL to 1 × 10^8^ IU/mL.

The Cobas TaqMan assay was performed at the Nanfang Hospital according to the manufacturer’s instructions (Roche Molecular Systems, Inc, Pleasanton, CA, USA). The Cobas TaqMan assay is based on the co-amplification of target HBV DNA and the detection of a cleaved dual-labeled oligonucleotide detection probe specific to the target. Serum samples of 750 μL were transferred for automatic processing. The manufacturer reports a HBV DNA linear range of 20 IU/mL to 1.7 × 10^8^ IU/mL.

The HBV genotype was determined by nested PCR with the Qiagen Mini Kit (Qiagen), following the manufacturer’s instructions.

### Follow-up

The enrolled patients were seen from July 2011 to September 2012 in 1 of 4 teaching hospitals—Nanfang Hospital of Southern Medical University in Guangzhou (43 patients), the Second Xiangya Hospital of Central South University in Changsha (13 patients), the Affiliated Sir Run Run Shaw Hospital of Zhejiang University in Hangzhou (3 patients), or Southwest Hospital of the Third Military Medical University in Chongqing (8 patients). Serum ALT (Olympus AU5400) and HBeAg levels (radioimmunoassay) were assessed at week 12. At week 24, serum ALT, HBeAg, and HBV DNA levels were evaluated.

Virological responses were scored according to the guidelines recommended by the European Association for the Study of the Liver (EASL) for nucleos(t)ide analogues (NAs) therapy [6].

### Statistical analysis

Mean and standard deviation (SD) were calculated for continuous variables, and percentages were used for categorical variables. Levels of quantified HBV DNA levels were expressed in logarithmic units (log_10_ IU/mL). HBV DNA levels with the Cobas TaqMan were assigned as negative, if results were below 20 IU/mL and thus were considered to be *undetectable* values. To determine whether the results were statistically different, the χ^2^ test, the *t* test, and the Spearman correlation coefficient were applied, as appropriate. For each pair of measurements, the agreement between the Cobas TaqMan and the Daan test were plotted against their means in a Bland-Altman analysis. The statistical significance of all tests was defined as *P* < 0.05 by 2-tailed tests. Quality control procedures, as well as data analyses, were performed by using MedCalc for Windows, version 9.38 (MedCalc Software, Mariakerke, Belgium).

## Results

### Demographics

Sixty-seven treatment-naïve patients (48 male, 19 female) who were diagnosed with hepatitis B virus e antigen (HBeAg) positive CHB with high viral load at baseline from July 2011 to March 2012 were enrolled in a multicenter prospective trial. The mean age was 30.6 ± 8.4 years (range: 16 to 61 years) (Table 1). HBV genotype B was found in 43 patients (64.2%, all B2 subgenotype) and HBV genotype C was found in 24 patients (35.8%; 17 with subgenotype C1 and 7 with C2 subgenotype). Forty-one out of the 67 patients (61.2%) received oral antiviral treatment with de novo lamivudine (LAM) and adefovir (ADV) combination therapy and 26 patients received entecavir (ETV).

**Table 1:** Demographic and virologic characteristics of the study patients

	**n = 67**
Age (years)^a^	30.6 ± 8.4
Gender, n (%)	
Male	48 (71.6)
Female	19 (28.4)
Treatment, n (%)	
Lamivudine plus adefovir	41 (61.2)
Entecavir	26 (38.8)
ALT (IU/mL)^a^	202.9 ± 137.9
HBV DNA (log_10_ IU/mL)	
Daan Test^a,b^	7.11 ± 0.44
Cobas TaqMan^a,b^	7.90 ± 0.73

^a^The value is expressed as mean ± SD.

^b^The value is expressed after logarithmic transformation (log_10_ IU/mL).

### Virological responses

The results from the Daan test indicated that 34 patients (50.7%) achieved virological responses and 33 patients (49.3%) attained partial virological responses. The HBV DNA levels obtained by the Cobas TaqMan assay indicated that 8 patients (11.9%) patients had virological responses and 58 patients (86.6%) had partial virological responses. Thus, the clinical assessments of the 34 samples by the two assays were significantly different (P < 0.001). In particular, 1 study patient who was classified as a partial virological responder by the Daan Test was classified as a primary non-responder by the Cobas TaqMan assay. Therefore, this comparison indicates that the more sensitive assay can alter clinical decisions and is crucial for managing CHB patients.

### Comparison of assays for measuring high HBV viral load in clinical samples

The HBV DNA levels of the 67 serum samples at baseline showed a high viral load and were detected by both the Cobas TaqMan and the Daan tests. All 67 baseline serum samples were quantitated by the Daans test whereas 27 of 67 of these baseline samples needed to be diluted for quantification by the Cobas TaqMan test (17 samples had HBV B genotype and 10 samples C genotype). The differences in the viral load of the 27 samples ranged from < 0.5log_10_ IU/mL in 3 samples (11.1%) to between 0.5log_10_ IU/mL and 1log_10_ IU/mL in 10 samples (37.0%) to ≥1 log_10_ IU/mL in 14 samples (51.9%). Forty samples were measured without dilution by the Cobas TaqMan test (26 had B genotype and 14 had C genotype). Genotypes were not significantly different at baseline viral load (p = 0.87).

The mean baseline viral load of patients treated with LAM/ADV and assessed by Cobas TaqMan (8.02 ± 0.57log_10_ IU/mL) was significantly higher than those assessed by Daan test (7.01 ± 0.43log_10_ IU/mL; P < 0.001). Likewise, the mean baseline viral load of patients treated with ETV assessed with the Cobas TaqMan (7.71 ± 0.90log_10_ IU/mL) was significantly greater than those assessed by the Daan test (7.13 ± 0.46log_10_ IU/mL; P = 0.002). Thus, HBV DNA levels with the Cobas TaqMan were significantly greater than that of the Daan Test (7.90 ± 0.72 log_10_ IU/mL versus 7.11 ± 0.44 log_10_ IU/mL, respectively; *P* < 0.001). Of note, the level of high viral load (≥7 log_10_ IU/mL) detected by the Cobas TaqMan test (8.09 ± 0.43) significantly differed from that of the Daan test (7.14 ± 0.43) (*P* < 0.001).

### Comparison of assays for measuring low HBV viral load (week 24 posttreatment clinical samples)

Another 67 serum samples were obtained from the patients at week 24 post treatment: the HBV DNA levels of only 33 clinical samples were detected by both assays. No significant differences were observed between the average HBV DNA levels with the Cobas TaqMan assay (3.25 ± 1.37 log_10_ IU/mL; range, from 1.34 log_10_ IU/mL to 6.41 log_10_ IU/mL) of these 33 posttreatment samples and those determined with the Daan test (3.52 ± 0.97 log_10_ IU/mL; range, from 2.20 log_10_ IU/mL to 6.86 log_10_ IU/mL) (*P* = 0.19). The correlation coefficient for the HBV DNA levels in these 33 samples detected by these two assays was 0.56 (Spearman correlation, *P* = 0.001). The divergence in the HBV DNA levels between both assays was within 0.5 log_10_ IU/mL and was found in only 10 samples.

The Cobas TaqMan test detected HBV DNA levels in 59 of the 67 week 24 serum samples (88.1%) whereas the Daan test detected HBV DNA levels in 33 of the 67 week 24 serum samples (49.7%)( *P* < 0.001). Thus, the Cobas TaqMan test quantitated HBV DNA levels in 26 of the 34 serum samples that had HBV DNA levels below the linear range of Daans test. The minimal value of these 26 samples was 1.44log_10_ IU/mL, the maximal value was 3.66log_10_ IU/mL, and the mean was 2.15 ± 0.58 log IU/mL.

The 24 week patient samples that tested positive by Cobas TaqMan showed no marked difference in HBV genotypes: HBV genotype B was present in 83.72% of samples and C in 95.83% samples, (*P* = 0.14). The patient samples that tested positive by Daans also showed no significant difference among genotypes: 44.2% of samples contained HBV genotypes B and 58.33% contained HBV genotype C (*P* = 0.26). Multivariate Analysis of Variance (MANOVA) was employed to analyze the HBV DNA determined by Cobas TaqMan assay and Daan test. Results showed HBV genotypes had no influence on the HBV DNA level (*P* = 0.471).

The mean reduction of viral load from baseline to samples after 24 week treatment with LAM/ADV or ETV was not significantly different between the two assays. The mean reduction of viral load in LAM/ADV group by the Cobas TaqMan (5.47 ± 1.53log IU/mL) was similar to that by the Daan test (5.32 ± 1.96log_10_ IU/mL; P = 0.57). The mean reductions of viral load in the ETV group after 24 weeks assessed by the Cobas TaqMan (5.20 ± 1.17log_10_ IU/mL) was similar to that assessed by the Daan test (29 ± 1.83log_10_ IU/mL; P = 0.8).

### Association analyses between the Cobas TaqMan assay and the Daan test

**Figure 1:**
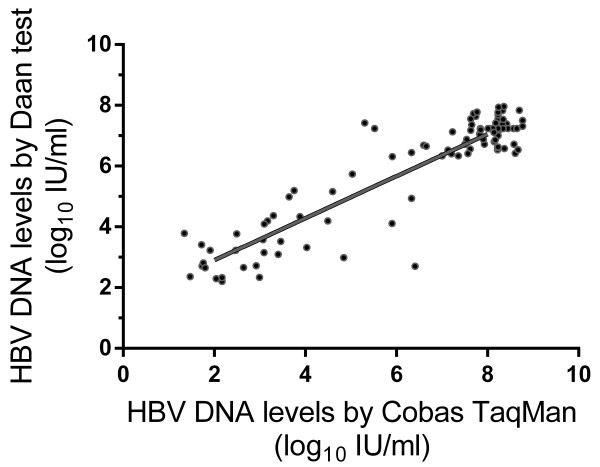
**The correlation of 2 methods in a total of the hundred positive of the 134 samples.** The Spearman’s correlation coefficient in HBV DNA level between the 2 methods was *r*_*p*_ = 0.79 (*P* < 0.001).

The HBV DNA levels of the 100 positive samples of the 134 (67 at baseline; 67 at week 24) samples detected by the Daan test were well correlated to those detected by the Cobas TaqMan (*r*_*p*_ = 0.79; *P* < 0.001; Figure 1). The reductions in viral load after 24 weeks of oral antiviral treatment in the 33 samples that were positive for both the Daan and the Cobas TaqMan tests were significantly different (3.59 ± 1.11 log_10_ IU/mL versus 4.87 ± 1.58 log_10_ IU/mL, respectively; *P* = 0.001). Spearman correlation analysis showed positive correlation between results from two tests (r_p_ = 0.602,P<0.001). However, according to the results from the Daan test, 34 patients (34/67; 50.7%) had undetectable HBV DNA levels, and 33 patients (33/67, 49.3%) had decreases in HBV DNA levels of more than 1 log_10_ IU/mL from baseline (but were still detectable) after 24 weeks on treatment. In comparison, the results of the Cobas TaqMan test indicated that 8 patients (8/67; 11.9%) had undetectable HBV DNA levels, and 58 patients (58/67; 86.6%) showed decreases in HBV DNA levels of more than 1 log_10_ IU/mL (but were still detectable) after 24 weeks on therapy (*P* < 0.001). A reduction of HBV viral load in one patient (1/67; 1.5%) varied from 1.38 log_10_ IU/mL with the Daan Test to –0.42 log_10_ IU/mL with the Cobas TaqMan.

### Agreement analyses between the Cobas TaqMan assay and the Daan test

**Figure 2:**
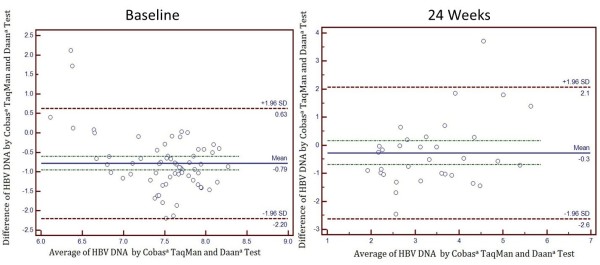
A Bland–Altman plot of the Cobas TaqMan assay and Daan test at baseline and 24 weeks post treatment.

A Bland-Altman plot was used to visualize the differences between HBV DNA quantitation of both baseline and posttreatment serum samples (n = 134) by the two methods (Figure 2). At baseline, the mean difference between the Cobas TaqMan assay and the Daan test was −0.79 log_10_ IU/mL, with limits of agreement from −2.20 log_10_ IU/mL to 0.63 log_10_ IU/mL. The HBV DNA levels in the baseline high viral loads (>10^6^ IU/mL) determined with the Daan test were lower than those determined by the Cobas Taqman test. In contrast, at 24-week postteatment, the Cobas TaqMan and the Daan test showed good agreement on low viral load HBV DNA levels, with the mean difference of –0.3 log_10_ IU/mL and with limits of agreement from −2.6 log_10_ IU/mL to 2.1 log_10_ IU/mL.

## Discussion

The HBV DNA levels from the 134 samples detected by both the Daan test and Cobas TaqMan were, in general, concordant. The HBV genotypes and the anti-viral therapy did not significantly affect the measurement of the viral loads by either the Daan or the Cobas Taqman assays. However, through further stratified analyses that accounted for the significant differences in the high viral load samples (>7 log_10_ IU/mL), we conclude that the Cobas TaqMan assay was superior to that of the Daan test. Therefore, it might be necessary to reconsider the usage of the Daan test because the Daan test was not as sensitive in detecting and quantifying HBV DNA levels as the Cobas TaqMan test in our study.

Serum HBV DNA levels are key indicators for CHB management, including assessing treatment initiation, treatment response, and drug withdrawal [6,18]. A more effective and sensitive method to detect HBV DNA levels could assist in improving key medical decisions and clinical patient management [19]. Current studies have demonstrated that monotherapy with a high resistance barrier agent or de novo combination therapy is a more appropriate choice for CHB patients with high baseline HBV DNA levels, as the risk of resistance and treatment failure are associated with high viral loads [20]. In order to avoid the emergence of drug resistant HBV [21,22], HBV DNA levels should be confirmed as early as possible. According to the results of the current study, viral load monitoring and HBV DNA detection were superior using the Cobas TaqMan assay to identify a virological response, a partial virological response, or a primary non-response.

Drug withdrawal is another key problem in CHB management. According to the most recently proposed guidelines, treatment withdrawal could be considered if the HBV DNA levels remained undetectable for at least 12 months, as documented on 2 separate occasions, which were 6 months apart, in HBeAg-positive patients. The treatment for HBeAg-negative patients would be continued for at least 18 months, if undetectable HBV DNA levels could be documented on 3 separate occasions, which were 6 months apart [6,18,23]. Due to the significant differences in the undetectable rates in the low viral load samples between the Daan test and the Cobas TaqMan assay (50.7% versus 11.9% respectively, *P* < 0.001), we believe it is necessary to measure the HBV DNA levels with the Cobas TaqMan assay to further confirm if the HBV DNA levels are negative, even if the HBV DNA levels were undetectable by the Daan test.

This study had a few limitations. This study had a small sample size, which may limit the generalizability of the current findings. Further research which utilizes statistical analyses to determine an appropriate sample size for sufficient statistical power is warranted to validate the current findings. Additionally, we only examined the HBV DNA levels at baseline and at 24 weeks. As such, we suggest additional examinations using these 2 tests at different times with larger sample sizes. Furthermore, more comprehensive studies with multiple detections by these same 2 kits are needed to confirm reproducibility and to determine if the Cobas TaqMan assay may deserve a greater priority than that of the Daan Test. Because the cost of the Cobas Taqman (500 yen) is greater than the Daan test (160 yen), a cost-benefit analysis also would be useful.

## Conclusions

Our results show that the Cobas TaqMan assay was more sensitive in detecting viral loads than the Daan test. Therefore, we do not think physicians should replace the Cobas TaqMan assay with the Daan real-time PCR test in terms of accurate quantitation. In particular, at moments of critical decisions, such as treatment initiation, switch of regimens, judgment of treatment efficacy and treatment cessation, the Cobas TaqMan test should be the first choice. This is because in some cases, the HBV DNA level may be lower than the detection limit of the Daan test, and the change from complete to partial virological response can affect clinical decisions.

However, given the low cost of the Daan test, physicians may consider using this method to monitor disease progression of patients during the immune-tolerant phase and of HBeAg positive patients for their mid to high HBV DNA levels.

## Abbreviations

HBV: Hepatitis B virus; CHB: Chronic hepatitis B; Daan test: Daan real-time polymerase chain reaction test; Cobas TaqMan: COBAS TaqMan HBV DNA Test; HCC: Hepatocellular carcinoma; HBsAg: HBV surface antigen; HBeAg: Hepatitis B virus e antigen; PCR: Polymerase chain reaction; ALT: Alanine aminotransferase; ULN: Upper limit of normal; HIV: Human immunodeficiency virus; LAM: Lamivudine; ADV: Adefovir; ETV: Entecavir; dNTPs: Deoxyribonucleoside triphosphates; SD: Standard deviation; NAs: Nucleos(t)ide analogues; EASL: European association for the study of the liver.

## Competing interests

The authors declare that they have no competing interests.

## Authors’ contributions

We declare that all the listed authors have participated actively in the study and all meet the requirements of the authorship. JP conceived and designed the study, and wrote the protocol, SHC managed the literature searches and analyses, undertook the statistical analysis, and wrote the first draft of the manuscript. FFL, YHZ and YGJ conducted patient follow-up, and provided important research specimens. All authors read and approve the manuscript.

## Pre-publication history

The pre-publication history for this paper can be accessed here:

http://www.biomedcentral.com/1471-2334/14/85/prepub
